# Crystal structure of bis­{2-[bis­(2-hy­droxy­eth­yl)amino]­ethanol-κ^3^ *O*,*N*,*O*′}zinc terephthalate

**DOI:** 10.1107/S1600536814021771

**Published:** 2014-10-11

**Authors:** Ya-Ping Li, Hu Zang, Dajun Sun, Julia Ming, Guan-Fang Su

**Affiliations:** a Department of Ophthalmology, The Second Hospital of Jilin University, 218 Ziqiang Street, Changchun 130041, People’s Republic of China; b Department of Orthopedics, The China–Japan Union Hospital of Jilin University Changchun, Changchun 130033, People’s Republic of China; c Department of Vascular Surgery, The China–Japan Union Hospital of Jilin University, Changchun 130033, People’s Republic of China; d St Erik’s Eye Hospital, Karolinska Institutet, Polhemsgatan 50, SE-112 82 Stockholm, Sweden

**Keywords:** crystal structure, chelate, hydrogen bonding, terephthalate

## Abstract

In the title salt, [Zn(C_6_H_15_NO_3_)_2_](C_8_H_4_O_4_), the Zn^II^ cation, located on a centre of inversion, is coordinated by four O atoms and two N atoms from two tridentate 2-[bis­(2-hy­droxy­eth­yl)amino]­ethanol (BHEA) ligands, giving rise to a slightly distorted octa­hedral geometry. The terephthalate dianion, located about a centre of inversion, is not coordinated to Zn^II^ but is connected through O—H⋯O contacts with [Zn(BHEA)_2_]^2+^ cations, leading to a three-dimensional crystal structure.

## Related literature   

For background and a related structure, see: Hamamci *et al.* (2002 ▶).
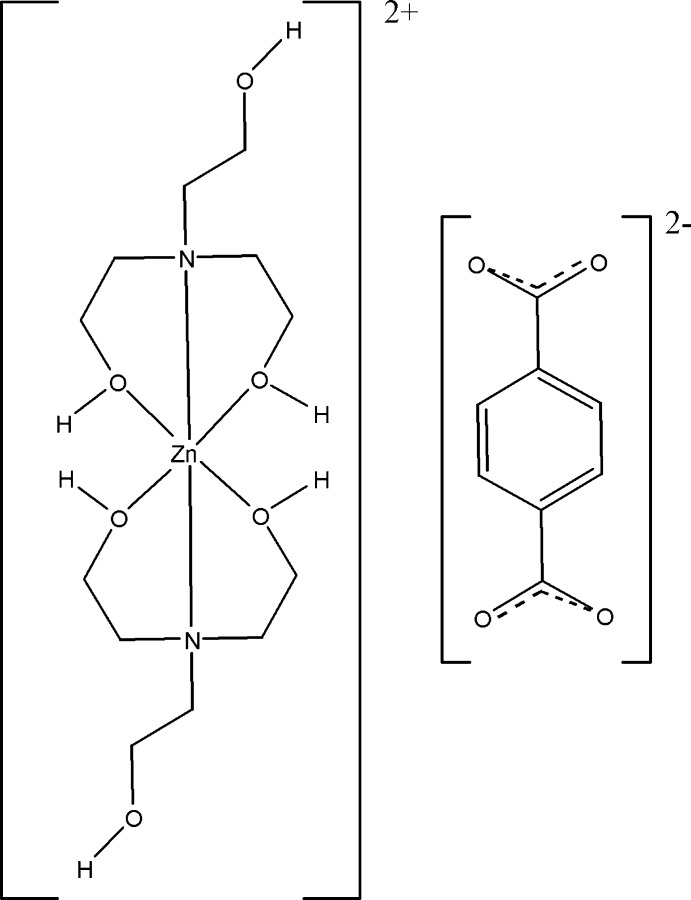


## Experimental   

### Crystal data  

[Zn(C_6_H_15_NO_3_)_2_](C_8_H_4_O_4_)
*M* *_r_* = 527.86
Triclinic, 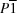

*a* = 7.963 (5) Å
*b* = 8.823 (5) Å
*c* = 9.198 (5) Å
α = 89.315 (5)°
β = 72.421 (5)°
γ = 66.208 (5)°
*V* = 559.2 (6) Å^3^
*Z* = 1
Mo *K*α radiation
μ = 1.16 mm^−1^
*T* = 293 K
0.26 × 0.24 × 0.23 mm

### Data collection  

Bruker SMART APEXII CCD diffractometer
Absorption correction: multi-scan (*SADABS*; Bruker, 2002 ▶) *T*_min_ = 0.725, *T*_max_ = 0.803
3145 measured reflections
2189 independent reflections
2165 reflections with *I* > 2σ(*I*)
*R*_int_ = 0.012

### Refinement  

*R*[*F* ^2^ > 2σ(*F* ^2^)] = 0.027
*wR*(*F* ^2^) = 0.072
*S* = 1.10
2189 reflections
160 parameters
3 restraints
H atoms treated by a mixture of independent and constrained refinement
Δρ_max_ = 0.45 e Å^−3^
Δρ_min_ = −0.51 e Å^−3^

### 

Data collection: *APEX2* (Bruker, 2002 ▶); cell refinement: *SAINT* (Bruker, 2002 ▶); data reduction: *SAINT*; program(s) used to solve structure: *SHELXS97* (Sheldrick, 2008 ▶); program(s) used to refine structure: *SHELXL97* (Sheldrick, 2008 ▶); molecular graphics: *SHELXTL* (Sheldrick, 2008 ▶); software used to prepare material for publication: *SHELXTL* and *publCIF* (Westrip, 2010 ▶).

## Supplementary Material

Crystal structure: contains datablock(s) global, I. DOI: 10.1107/S1600536814021771/tk5343sup1.cif

**Fig. 1.**
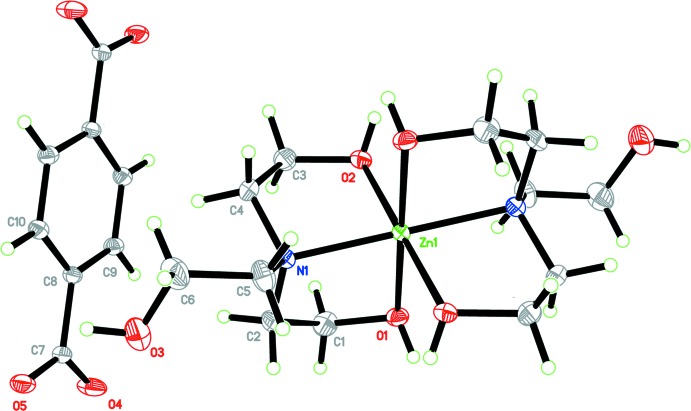
A view of the ions in the title salt. Displacement ellipsoids are drawn at the 30% probability level. Unlabelled atoms in the cation are related by 1-x, -y, -z, and those in the dianion by 1-x, -1-y, 1-z.

Structure factors: contains datablock(s) I. DOI: 10.1107/S1600536814021771/tk5343Isup2.hkl

x y z x y z . DOI: 10.1107/S1600536814021771/tk5343fig1.tif
A view of the ions in the title salt. Displacement ellipsoids are drawn at the 30% probability level. Unlabelled atoms in the cation are related by 1-*x*, −*y*, −*z*, and those in the dianion by 1-*x*, −1-*y*, 1-*z*.

**Fig. 2.**
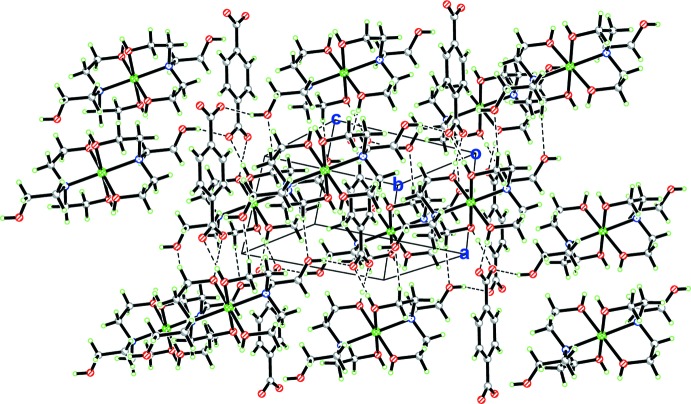
A view of the crystal structure of the title salt. Hydrogen bonds are shown as dashed lines.

. DOI: 10.1107/S1600536814021771/tk5343fig2.tif
A view of the crystal structure of the title salt. Hydrogen bonds are shown as dashed lines.

CCDC reference: 1027329

Additional supporting information:  crystallographic information; 3D view; checkCIF report

## Figures and Tables

**Table 1:** Hydrogen-bond geometry (, )

*D*H*A*	*D*H	H*A*	*D* *A*	*D*H*A*
O1H1O5^i^	0.82(2)	1.82(2)	2.632(2)	177(2)
O2H2O4^ii^	0.83(2)	1.74(2)	2.564(2)	178(2)
O3H3O5^iii^	0.87(2)	2.13(2)	2.942(3)	155(2)

Symmetry codes: (i) 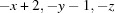; (ii) 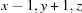; (iii) 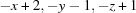.

## supplementary crystallographic information

### S1. Preparation

The synthesis was performed under hydro­thermal conditions. A mixture of Zn(CH_3_COO)_2_^.^2(H_2_O), (0.2 mmol, 0.044 g), 2-[bis­(2-hy­droxy­ethyl)­amino]­ethanol (0.4 mmol, 0.062 g), sodium terephthalate (0.2 mmol, 0.042 g) and H_2_O (20 mL) in a 30 mL stainless steel reactor with a Teflon liner was heated from 293 to 433 K in 2 h and then held at a constant temperature of 433 K for 72 h, after which the mixture was cooled to 298 K. Colourless crystals of the title compound were recovered from the reaction.

### S2. Refinement

All C-bound H atoms were positioned with idealized geometry (0.93–0.97 Å) and refined isotropically with *U*_iso_(H) = 1.2 *U*_eq_(C) using a riding model. The hy­droxy H-atoms were located in a different Fourier map and were refined with an O—H distance restrained to 0.85 (2) Å and with *U*_iso_(H) = 1.5 *U*_eq_(O).

### Crystal data

**Table:**

[Zn(C_6_H_15_NO_3_)_2_](C_8_H_4_O_4_)	*Z* = 1
*M**_r_* = 527.86	*F*(000) = 278
Triclinic, *P*1	*D*_x_ = 1.567 Mg m^−^^3^
Hall symbol: -P 1	Mo *K*α radiation, λ = 0.71069 Å
*a* = 7.963 (5) Å	Cell parameters from 2165 reflections
*b* = 8.823 (5) Å	θ = 1.7–22.8°
*c* = 9.198 (5) Å	µ = 1.16 mm^−^^1^
α = 89.315 (5)°	*T* = 293 K
β = 72.421 (5)°	Block, colourless
γ = 66.208 (5)°	0.26 × 0.24 × 0.23 mm
*V* = 559.2 (6) Å^3^	

### Data collection

**Table:**

Bruker SMART APEXII CCD diffractometer	2189 independent reflections
Radiation source: fine-focus sealed tube	2165 reflections with *I* > 2σ(*I*)
Graphite monochromator	*R*_int_ = 0.012
φ and ω scans	θ_max_ = 26.2°, θ_min_ = 2.3°
Absorption correction: multi-scan (*SADABS*; Bruker, 2002)	*h* = −9→8
*T*_min_ = 0.725, *T*_max_ = 0.803	*k* = −10→10
3145 measured reflections	*l* = −10→11

### Refinement

**Table:**

Refinement on *F*^2^	Primary atom site location: structure-invariant direct methods
Least-squares matrix: full	Secondary atom site location: difference Fourier map
*R*[*F*^2^ > 2σ(*F*^2^)] = 0.027	Hydrogen site location: inferred from neighbouring sites
*wR*(*F*^2^) = 0.072	H atoms treated by a mixture of independent and constrained refinement
*S* = 1.10	*w* = 1/[σ^2^(*F*_o_^2^) + (0.0381*P*)^2^ + 0.3177*P*] where *P* = (*F*_o_^2^ + 2*F*_c_^2^)/3
2189 reflections	(Δ/σ)_max_ < 0.001
160 parameters	Δρ_max_ = 0.45 e Å^−^^3^
3 restraints	Δρ_min_ = −0.51 e Å^−^^3^

### Special details

**Table:**

Geometry. All esds (except the esd in the dihedral angle between two l.s. planes) are estimated using the full covariance matrix. The cell esds are taken into account individually in the estimation of esds in distances, angles and torsion angles; correlations between esds in cell parameters are only used when they are defined by crystal symmetry. An approximate (isotropic) treatment of cell esds is used for estimating esds involving l.s. planes.
Refinement. Refinement of F^2^ against ALL reflections. The weighted R-factor wR and goodness of fit S are based on F^2^, conventional R-factors R are based on F, with F set to zero for negative F^2^. The threshold expression of F^2^ > 2sigma(F^2^) is used only for calculating R-factors(gt) etc. and is not relevant to the choice of reflections for refinement. R-factors based on F^2^ are statistically about twice as large as those based on F, and R- factors based on ALL data will be even larger.

### Fractional atomic coordinates and isotropic or equivalent isotropic displacement parameters (Å^2^)

**Table:**

	*x*	*y*	*z*	*U*_iso_*/*U*_eq_	
C1	0.6774 (3)	−0.3639 (2)	−0.0601 (2)	0.0370 (5)	
H1A	0.7747	−0.4664	−0.1248	0.044*	
H1B	0.5597	−0.3796	−0.0138	0.044*	
C2	0.7484 (3)	−0.3279 (2)	0.0655 (2)	0.0331 (4)	
H2A	0.7475	−0.4087	0.1378	0.040*	
H2B	0.8814	−0.3417	0.0199	0.040*	
C3	0.3010 (3)	−0.1603 (3)	0.2345 (2)	0.0363 (5)	
H3A	0.3374	−0.2759	0.1998	0.044*	
H3B	0.1832	−0.1228	0.3224	0.044*	
C4	0.4611 (3)	−0.1470 (3)	0.2818 (2)	0.0325 (4)	
H4A	0.4091	−0.0417	0.3462	0.039*	
H4B	0.5057	−0.2361	0.3426	0.039*	
C5	0.7460 (3)	−0.0863 (3)	0.2013 (3)	0.0371 (5)	
H5A	0.6741	0.0341	0.2208	0.044*	
H5B	0.8648	−0.1107	0.1168	0.044*	
C6	0.8006 (4)	−0.1462 (3)	0.3430 (3)	0.0463 (5)	
H6A	0.8579	−0.0793	0.3745	0.056*	
H6B	0.6851	−0.1329	0.4268	0.056*	
C7	0.9062 (2)	−0.7092 (2)	0.3337 (2)	0.0260 (4)	
C8	0.6957 (2)	−0.5992 (2)	0.4202 (2)	0.0228 (3)	
C9	0.5509 (3)	−0.6072 (2)	0.3698 (2)	0.0253 (4)	
H9	0.5846	−0.6789	0.2823	0.030*	
C10	0.6436 (3)	−0.4910 (2)	0.5508 (2)	0.0250 (4)	
H10	0.7392	−0.4844	0.5852	0.030*	
N1	0.6286 (2)	−0.15730 (18)	0.14967 (17)	0.0229 (3)	
O1	0.63918 (19)	−0.23022 (16)	−0.15252 (15)	0.0273 (3)	
O2	0.26592 (18)	−0.06108 (17)	0.11409 (15)	0.0283 (3)	
O3	0.9319 (3)	−0.3128 (2)	0.3114 (2)	0.0553 (5)	
O4	0.9393 (2)	−0.8061 (2)	0.21957 (18)	0.0411 (4)	
O5	1.03466 (19)	−0.69733 (19)	0.37935 (16)	0.0357 (3)	
Zn1	0.5000	0.0000	0.0000	0.01988 (10)	
H1	0.738 (3)	−0.249 (3)	−0.224 (2)	0.030*	
H2	0.160 (3)	0.020 (2)	0.150 (3)	0.030*	
H3	0.954 (3)	−0.343 (3)	0.397 (2)	0.030*	

### Atomic displacement parameters (Å^2^)

**Table:**

	*U*^11^	*U*^22^	*U*^33^	*U*^12^	*U*^13^	*U*^23^
C1	0.0491 (12)	0.0214 (9)	0.0346 (10)	−0.0125 (9)	−0.0086 (9)	−0.0021 (8)
C2	0.0334 (10)	0.0202 (9)	0.0327 (10)	−0.0003 (7)	−0.0084 (8)	0.0034 (7)
C3	0.0277 (9)	0.0398 (11)	0.0383 (11)	−0.0150 (9)	−0.0056 (8)	0.0149 (9)
C4	0.0298 (9)	0.0384 (10)	0.0218 (9)	−0.0095 (8)	−0.0049 (7)	0.0076 (8)
C5	0.0427 (11)	0.0357 (11)	0.0466 (12)	−0.0202 (9)	−0.0284 (10)	0.0128 (9)
C6	0.0430 (12)	0.0505 (14)	0.0519 (14)	−0.0171 (11)	−0.0281 (11)	0.0066 (11)
C7	0.0202 (8)	0.0259 (9)	0.0237 (8)	−0.0041 (7)	−0.0038 (7)	0.0040 (7)
C8	0.0184 (8)	0.0208 (8)	0.0238 (8)	−0.0053 (6)	−0.0038 (6)	0.0041 (6)
C9	0.0231 (8)	0.0249 (9)	0.0233 (8)	−0.0071 (7)	−0.0055 (7)	−0.0011 (6)
C10	0.0205 (8)	0.0271 (9)	0.0263 (9)	−0.0084 (7)	−0.0082 (7)	0.0027 (7)
N1	0.0215 (7)	0.0215 (7)	0.0236 (7)	−0.0066 (6)	−0.0078 (6)	0.0031 (6)
O1	0.0249 (6)	0.0268 (6)	0.0237 (6)	−0.0075 (5)	−0.0038 (5)	−0.0028 (5)
O2	0.0189 (6)	0.0293 (7)	0.0303 (7)	−0.0059 (5)	−0.0050 (5)	0.0047 (5)
O3	0.0529 (10)	0.0576 (11)	0.0483 (10)	−0.0096 (9)	−0.0261 (9)	0.0130 (8)
O4	0.0226 (7)	0.0423 (8)	0.0432 (8)	−0.0025 (6)	−0.0049 (6)	−0.0157 (7)
O5	0.0188 (6)	0.0481 (9)	0.0311 (7)	−0.0068 (6)	−0.0056 (5)	−0.0038 (6)
Zn1	0.01853 (15)	0.01801 (15)	0.01999 (15)	−0.00478 (11)	−0.00604 (10)	0.00264 (10)

### Geometric parameters (Å, º)

**Table:**

C1—O1	1.431 (2)	C6—H6B	0.9700
C1—C2	1.518 (3)	C7—O4	1.255 (2)
C1—H1A	0.9700	C7—O5	1.257 (2)
C1—H1B	0.9700	C7—C8	1.511 (2)
C2—N1	1.483 (2)	C8—C10	1.390 (3)
C2—H2A	0.9700	C8—C9	1.393 (3)
C2—H2B	0.9700	C9—C10^i^	1.390 (3)
C3—O2	1.427 (2)	C9—H9	0.9300
C3—C4	1.511 (3)	C10—C9^i^	1.390 (3)
C3—H3A	0.9700	C10—H10	0.9300
C3—H3B	0.9700	N1—Zn1	2.1282 (16)
C4—N1	1.480 (2)	O1—Zn1	2.1529 (16)
C4—H4A	0.9700	O1—H1	0.815 (16)
C4—H4B	0.9700	O2—Zn1	2.1169 (16)
C5—N1	1.496 (2)	O2—H2	0.825 (16)
C5—C6	1.519 (3)	O3—H3	0.870 (16)
C5—H5A	0.9700	Zn1—O2^ii^	2.1169 (16)
C5—H5B	0.9700	Zn1—N1^ii^	2.1282 (16)
C6—O3	1.388 (3)	Zn1—O1^ii^	2.1529 (16)
C6—H6A	0.9700		
			
O1—C1—C2	111.27 (16)	O5—C7—C8	118.64 (16)
O1—C1—H1A	109.4	C10—C8—C9	119.17 (16)
C2—C1—H1A	109.4	C10—C8—C7	121.09 (16)
O1—C1—H1B	109.4	C9—C8—C7	119.74 (16)
C2—C1—H1B	109.4	C10^i^—C9—C8	120.58 (17)
H1A—C1—H1B	108.0	C10^i^—C9—H9	119.7
N1—C2—C1	112.93 (16)	C8—C9—H9	119.7
N1—C2—H2A	109.0	C9^i^—C10—C8	120.26 (17)
C1—C2—H2A	109.0	C9^i^—C10—H10	119.9
N1—C2—H2B	109.0	C8—C10—H10	119.9
C1—C2—H2B	109.0	C4—N1—C2	113.25 (15)
H2A—C2—H2B	107.8	C4—N1—C5	109.91 (16)
O2—C3—C4	110.59 (16)	C2—N1—C5	112.28 (16)
O2—C3—H3A	109.5	C4—N1—Zn1	104.11 (11)
C4—C3—H3A	109.5	C2—N1—Zn1	108.27 (12)
O2—C3—H3B	109.5	C5—N1—Zn1	108.59 (12)
C4—C3—H3B	109.5	C1—O1—Zn1	107.34 (11)
H3A—C3—H3B	108.1	C1—O1—H1	108.2 (17)
N1—C4—C3	113.26 (16)	Zn1—O1—H1	120.1 (16)
N1—C4—H4A	108.9	C3—O2—Zn1	112.52 (11)
C3—C4—H4A	108.9	C3—O2—H2	108.0 (16)
N1—C4—H4B	108.9	Zn1—O2—H2	114.6 (16)
C3—C4—H4B	108.9	C6—O3—H3	106.6 (15)
H4A—C4—H4B	107.7	O2^ii^—Zn1—O2	180.00 (4)
N1—C5—C6	117.31 (18)	O2^ii^—Zn1—N1^ii^	81.99 (7)
N1—C5—H5A	108.0	O2—Zn1—N1^ii^	98.01 (7)
C6—C5—H5A	108.0	O2^ii^—Zn1—N1	98.01 (7)
N1—C5—H5B	108.0	O2—Zn1—N1	81.99 (7)
C6—C5—H5B	108.0	N1^ii^—Zn1—N1	180.0
H5A—C5—H5B	107.2	O2^ii^—Zn1—O1^ii^	90.41 (6)
O3—C6—C5	110.1 (2)	O2—Zn1—O1^ii^	89.59 (6)
O3—C6—H6A	109.6	N1^ii^—Zn1—O1^ii^	82.73 (7)
C5—C6—H6A	109.6	N1—Zn1—O1^ii^	97.27 (7)
O3—C6—H6B	109.6	O2^ii^—Zn1—O1	89.59 (6)
C5—C6—H6B	109.6	O2—Zn1—O1	90.41 (6)
H6A—C6—H6B	108.2	N1^ii^—Zn1—O1	97.27 (7)
O4—C7—O5	124.72 (16)	N1—Zn1—O1	82.73 (7)
O4—C7—C8	116.64 (16)	O1^ii^—Zn1—O1	180.00 (12)
			
O1—C1—C2—N1	47.2 (2)	C3—O2—Zn1—N1	4.13 (13)
O2—C3—C4—N1	−43.7 (2)	C3—O2—Zn1—O1^ii^	−93.26 (13)
N1—C5—C6—O3	68.3 (3)	C3—O2—Zn1—O1	86.74 (13)
O4—C7—C8—C10	178.15 (17)	C4—N1—Zn1—O2^ii^	154.45 (12)
O5—C7—C8—C10	−2.2 (3)	C2—N1—Zn1—O2^ii^	−84.78 (12)
O4—C7—C8—C9	−1.1 (3)	C5—N1—Zn1—O2^ii^	37.38 (13)
O5—C7—C8—C9	178.52 (17)	C4—N1—Zn1—O2	−25.55 (12)
C10—C8—C9—C10^i^	−0.3 (3)	C2—N1—Zn1—O2	95.22 (12)
C7—C8—C9—C10^i^	179.03 (16)	C5—N1—Zn1—O2	−142.62 (13)
C9—C8—C10—C9^i^	0.3 (3)	C4—N1—Zn1—N1^ii^	−80 (100)
C7—C8—C10—C9^i^	−179.02 (16)	C2—N1—Zn1—N1^ii^	41 (100)
C3—C4—N1—C2	−72.9 (2)	C5—N1—Zn1—N1^ii^	163 (100)
C3—C4—N1—C5	160.62 (17)	C4—N1—Zn1—O1^ii^	63.01 (12)
C3—C4—N1—Zn1	44.47 (18)	C2—N1—Zn1—O1^ii^	−176.22 (12)
C1—C2—N1—C4	87.9 (2)	C5—N1—Zn1—O1^ii^	−54.06 (13)
C1—C2—N1—C5	−146.89 (18)	C4—N1—Zn1—O1	−116.99 (12)
C1—C2—N1—Zn1	−27.01 (19)	C2—N1—Zn1—O1	3.78 (12)
C6—C5—N1—C4	48.9 (2)	C5—N1—Zn1—O1	125.94 (13)
C6—C5—N1—C2	−78.1 (2)	C1—O1—Zn1—O2^ii^	118.61 (13)
C6—C5—N1—Zn1	162.17 (16)	C1—O1—Zn1—O2	−61.39 (13)
C2—C1—O1—Zn1	−40.83 (19)	C1—O1—Zn1—N1^ii^	−159.52 (12)
C4—C3—O2—Zn1	18.7 (2)	C1—O1—Zn1—N1	20.48 (12)
C3—O2—Zn1—O2^ii^	116 (100)	C1—O1—Zn1—O1^ii^	−79 (100)
C3—O2—Zn1—N1^ii^	−175.87 (13)		

Symmetry codes: (i) −*x*+1, −*y*−1, −*z*+1; (ii) −*x*+1, −*y*, −*z*.

### Hydrogen-bond geometry (Å, º)

**Table:**

*D*—H···*A*	*D*—H	H···*A*	*D*···*A*	*D*—H···*A*
O1—H1···O5^iii^	0.82 (2)	1.82 (2)	2.632 (2)	177 (2)
O2—H2···O4^iv^	0.83 (2)	1.74 (2)	2.564 (2)	178 (2)
O3—H3···O5^v^	0.87 (2)	2.13 (2)	2.942 (3)	155 (2)

Symmetry codes: (iii) −*x*+2, −*y*−1, −*z*; (iv) *x*−1, *y*+1, *z*; (v) −*x*+2, −*y*−1, −*z*+1.
