# Supplementary figures and images for: Crystal structure of bis­{2-[bis­(2-hy­droxy­eth­yl)amino]­ethanol-κ3 O,N,O′}zinc terephthalate
Source: Acta Crystallogr Sect E Struct Rep Online. 2014 Oct 11;70(Pt 11):m361–2. doi: 10.1107/S1600536814021771 (PMC4257296; doi:10.1107/S1600536814021771)

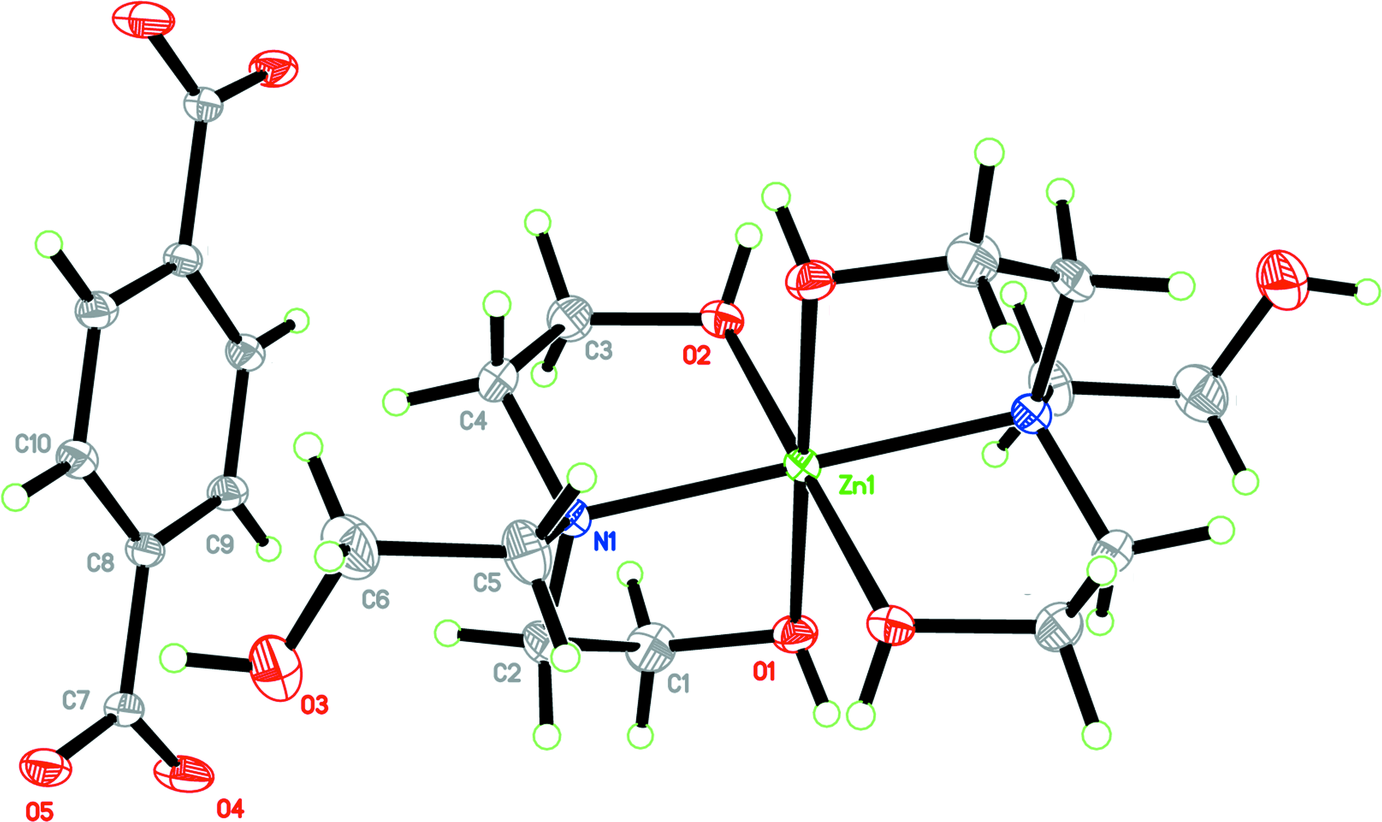

Supplement: Supplementary file 3 [file e-70-0m361-fig1.tif]

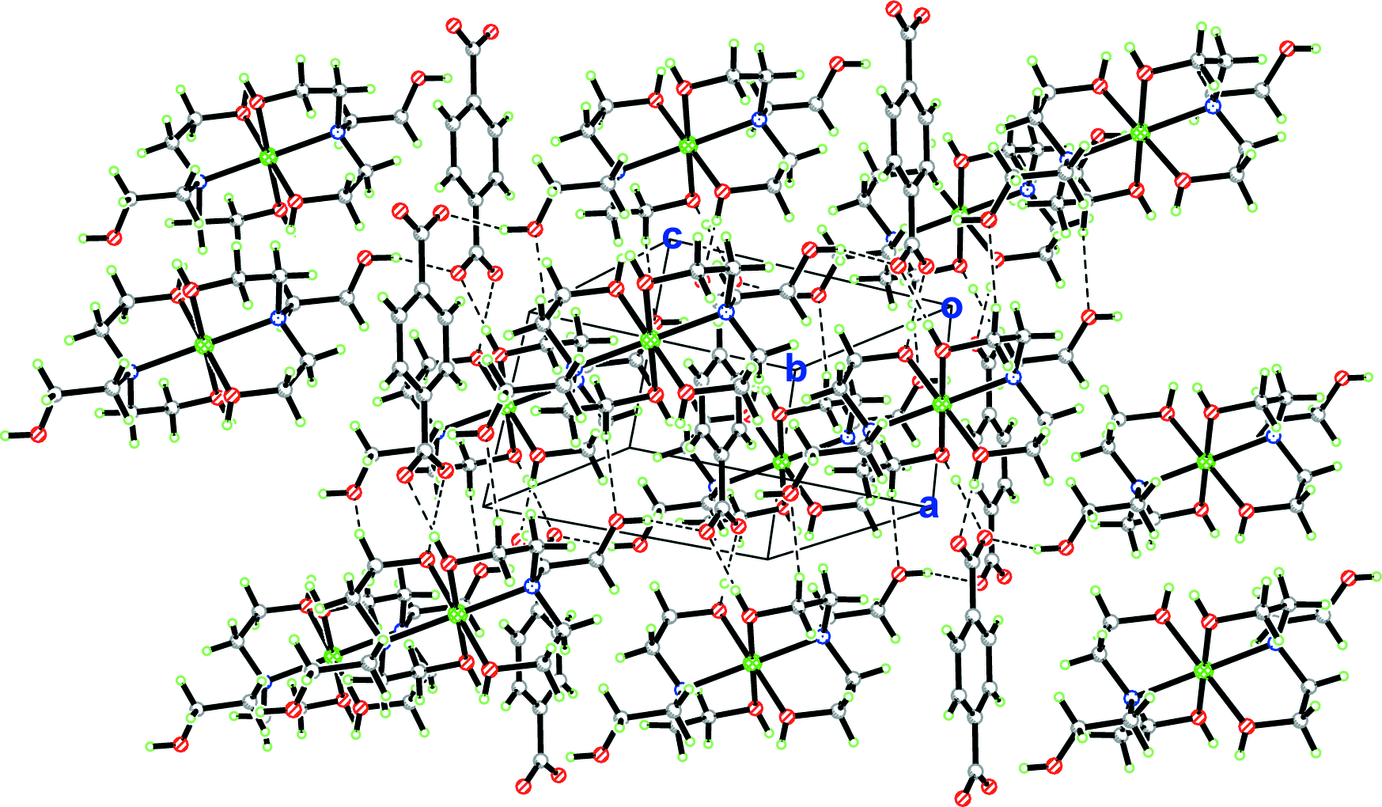

Supplement: Supplementary file 4 [file e-70-0m361-fig2.tif]
